# Alleles on locus chromosome 4B from different parents confer tiller number and the yield-associated traits in wheat

**DOI:** 10.1186/s12870-024-05079-4

**Published:** 2024-05-24

**Authors:** Yahui Li, Jinghuang Hu, Yunfeng Qu, Dan Qiu, Huailong Lin, Jiuyuan Du, Lu Hou, Lin Ma, Qiuhong Wu, Yang Zhou, Hongjun Zhang, Li Yang, Hongwei Liu, Zhiyong Liu, Yijun Zhou, Hongjie Li

**Affiliations:** 1 College of Life and Environmental Sciences, Minzu University of China, Beijing, 100081 China; 2 The National Engineering Laboratory of Crop Molecular Breeding, Institute of Crop Sciences, Chinese Academy of Agricultural Sciences, Beijing, 100081 China; 3 Institute of Genetics and Developmental Biology, Chinese Academy of Sciences, Beijing, 100101 China; 4 State Key Laboratory of Crop Stress Adaptation and Improvement, School of Life Sciences, Henan University, Kaifeng, 475001 China; 5 Jiushenghe Seed Industry Co. Ltd, Changji, 831100 China; 6 Wheat Research Institute, Gansu Academy of Agricultural Sciences, Lanzhou, 730070 China; 7 Key Laboratory of Agricultural Integrated Pest Management, Qinghai Academy of Agricultural and Forestry Sciences, Qinghai University, Xining, 810016 China; 8 Datong Hui and Tu Autonomous County Agricultural Technology Extension Center, Xining, 810100 China

**Keywords:** *Triticum aestivum*, Quantitative trait loci, Tiller number, Spike traits, Grain traits

## Abstract

Pleiotropy is frequently detected in agronomic traits of wheat (*Triticum aestivum*). A locus on chromosome 4B, *QTn/Ptn/Sl/Sns/Al/Tgw/Gl/Gw.caas-4B*, proved to show pleiotropic effects on tiller, spike, and grain traits using a recombinant inbred line (RIL) population of Qingxinmai × 041133. The allele from Qingxinmai increased tiller numbers, and the allele from line 041133 produced better performances of spike traits and grain traits. Another 52 QTL for the eight traits investigated were detected on 18 chromosomes, except for chromosomes 5D, 6D, and 7B. Several genes in the genomic interval of the locus on chromosome 4B were differentially expressed in crown and inflorescence samples between Qingxinmai and line 041133. The development of the KASP marker specific for the locus on chromosome 4B is useful for molecular marker-assisted selection in wheat breeding.

**Supplementary Information:**

The online version contains supplementary material available at 10.1186/s12870-024-05079-4.

## Introduction

Wheat (*Triticum aestivum* L.) is a long-historically grown crop in more than 40 countries. It feeds about 30% of the world’s population. Despite China, as a leading wheat grower, producing approximately 17% of the global wheat, the demand for wheat is increasing due to the population growth and rapid urbanization [1–3]. Increase in wheat yield per annum from the 1920s was estimated to be 1.29% and 1.50% for north and south winter wheat in China [4]. Nevertheless, this yield increment does not meet the future demand for wheat [5]. Further increase in grain yields is the highest priority in most wheat breeding programs throughout the country.

Wheat yield is determined by many agronomic traits, such as the number of spikes per unit area, grain numbers per spike (GNS), and thousand-grain weight (TGW) [6]. Because direct selection of grain yield is difficult, the improvement of yield-associated traits is often conducted instead. In fact, GNS and TGW have been increased in wheat cultivars released in the past several decades in China [4]. Many genetic loci governing grain weight were identified on different wheat chromosomes [7]. Several studies reported quantitative trait loci (QTL) for grain weight on the short arm of chromosome 4B. *QTKW.caas-4BS* for grain weight was mapped in a 483 kb genomic interval in Doumai, which harbors three genes *ZnF*, *EamA*, and *Rht-B1* [8]. The functions of *Rht-B1* (encoding gibberellin signaling repressor) and *ZnF-B* (encoding a RING-type E3 ligase) on grain yield were determined using natural deletion of a haploblock (∼ 500 kb) in wheat cultivar Heng597 [9]. Grain weight is associated with grain size measured by grain length (GL) and grain width (GW) [10–13]. Chen et al. (2020) [14] identified 30 stable QTL including *QTgw.cau-7D* and *QGw.cau-7D* for grain size on chromosome 7D.

Tiller is a branching phenomenon in monocotyledons [15, 16], which determines the number of spikes. A low-tillering gene, *tin1*, was localized on the short arm of wheat chromosome 1A [17, 18]. Several genes or QTL for increasing or decreasing tiller numbers were identified. Genes *tin3* and *TaD27* inhibit tiller formation [19, 20]. *QMTN.sicau-4D* and *QPtn.sau-4B* increase tiller numbers [21, 22]. *QTa.sau-2B-769* for tiller angle was detected on the chromosome arm 2BL. *TraesCS2B01G583800*, a gene regulating leaf angle, was most likely the candidate gene for this QTL [23].

A wheat spike is composed of spikelets borne on its rachis. Spike length (SL) is closely correlated with spikelet number (SN). Both traits are influenced by interaction between genetic and environmental factors. Li et al. [24] mapped two major QTL for SL, *QSc/Sl.cib-5A* and *QSc/Sl.cib-6 A*, explaining 7.13–33.6% of the phenotypic variations. *TaAPO-A1* confers total SN in European winter wheat cultivars [25]. *QSns.sau-2D* was detected on the chromosome arm 2DS and can explain 10.16–45.68% of phenotypic variations [26]. An important pleiotropic QTL, *Q.SpnN/SpkLng/PH/SPP.3A*, was identified on chromosome 3A. It was found to be associated with spikelet number per spike, spike length, plant height, and spikes per spike [27].

Some wheat cultivars have awns in glumes. Awn is an important organ for respiration and photosynthesis of spikes. It not only plays a role in protection and transmission of seeds, but also impacts on grain yield. Assimilates synthesized by wheat awns are transported to grains nearby [28]. It is believed that *B1*, *B2*, and *HD* are the main genes inhibiting the awn development. Locus *B1* (tipped 1) on chromosome 5AL regulates the phenotype of tip awns, and *B2* (tipped 2) on chromosome 6BL shortens the awn length [29, 30]. *Hd*, a dominant allele of *knotted 1* (*kn-1*) on the chromosome arm 4AS, regulates the hook-awn phenotype of wheat [31].

Wheat landrace Qingxinmai is featured by a large number of tillers. It can develop 30–40 spikes per plant. Breeding line 041133 develops large spikes and grains. Incorporation of favorable alleles of loci for tiller number, spike, and grain traits from Qingxinmai and line 041133 is an option to increase yield of wheat. Toward this end, dissection of QTL for the traits of interest is a prerequisite. A recombinant inbred line (RIL) population was developed from a cross between these genotypes. The purpose of this study was to unravel the genetic control of the agronomic traits with the aid of bulk segregant analysis-RNA-Seq (BSR-Seq), bulked segregant exome capture sequencing (BSE-Seq), and a wheat 16 K genotyping by target sequencing (GBTS) single nucleotide polymorphism (SNP) array.

## Results

### Phenotypic performances

**Fig. 1:**
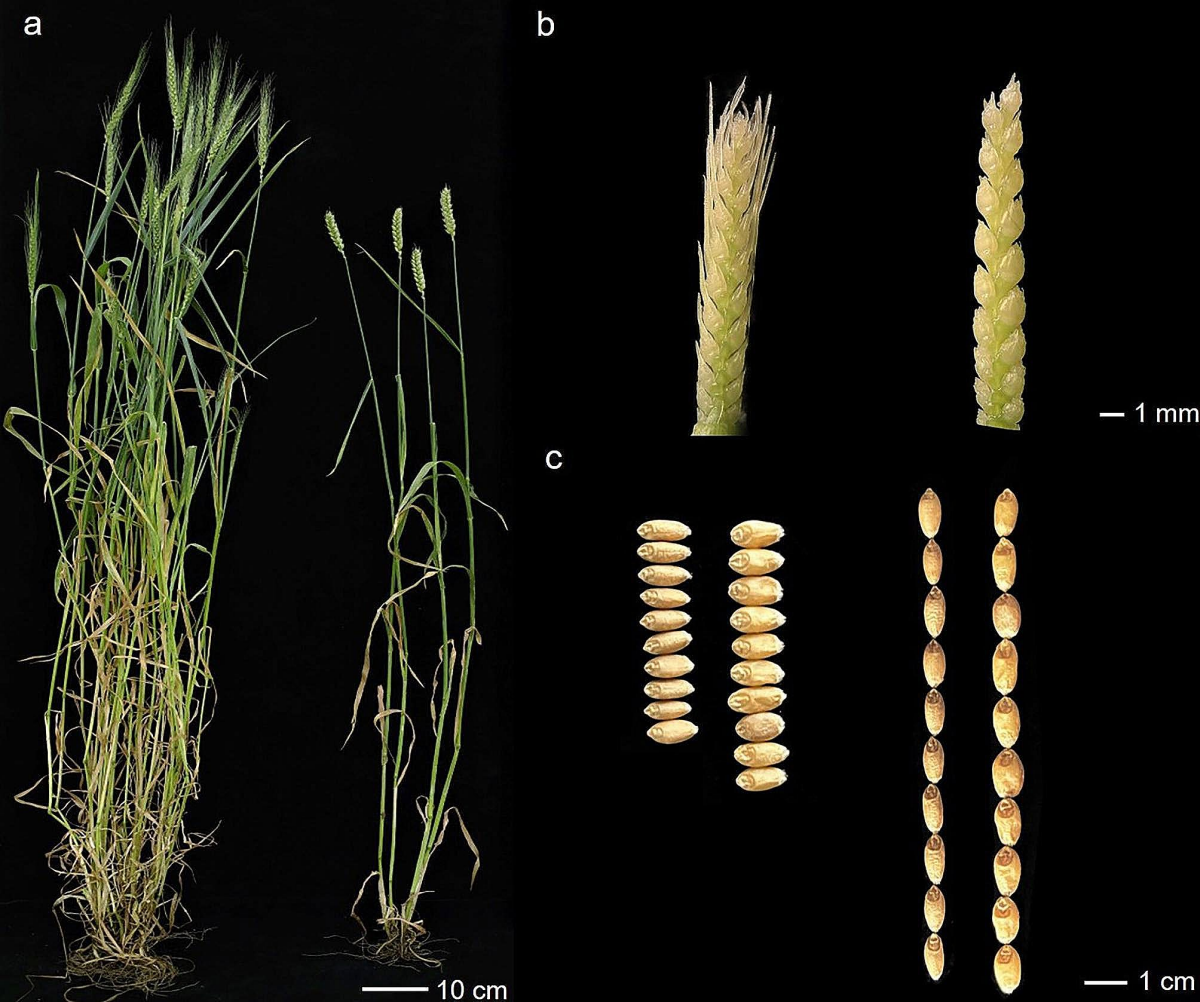
Plants (**a**), inflorescences (**b**), and grains (**c**) of Qingxinmai (left) and 041133 (right)

Qingxinmai and line 041133 differed significantly in tiller traits, total tiller number (TN) and productive tiller number (PTN), spike traits, spike length (SL), spikelet number per spike (SNS), and awn length (AL) (including top, central, and bottom positions on spikes), and grain traits, thousand-grain weight (TGW), grain length (GL), and grain width (GW) (*P* < 0.05). Qingxinmai had more tillers and longer awns, while line 041133 had longer spikes and larger kernels in different field trials (Fig. 1; Table 1). The broad-sense heritability (*H*^*2*^) for these traits ranged from 0.40 to 0.99.

**Table 1:** Phenotypic variation of total tiller number (TN), productive tiller number (PTN), awn length (AL), spike length (SL), spikelet number per spike (SNS), grain width (GW), grain length (GL), and thousand-grain weight (TGW) of the parents and the RIL population in different environments

Trait	Env				RIL lines				*H* ^*2*^
		Qingxinmai	41133	Range	Mean	Skewness	Kurtosis	CV (%)	
TN	2020XX	31.37	15.77	11.65–30.24	20.66 ± 3.23	0.31	0.13	15.63	0.40
	2020ZX	28.92	17.4	8.79–33.29	23.08 ± 4.64	–0.31	0.04	20.12	
	2020CP	21.39	8.16	4.33–29.52	14.51 ± 3.97	–0.21	2.36	27.41	
	BLUE	29.32	14.35	11.14–26.35	19.14 ± 2.40	0.06	0.27	12.53	
PTN	2020XX	26.19	10.25	8.7–33.37	18.32 ± 4.09	0.00	–0.39	22.31	0.73
	2020ZX	29.13	18.62	5.50–43.8	20.74 ± 4.94	0.08	(0.17)	23.81	
	2020CP	19.36	5.27	2.50–32.50	14.24 ± 3.54	0.29	1.36	24.86	
	BLUE	25.45	12.34	10.12–25.38	17.11 ± 3.19	–0.06	–0.49	18.61	
SL (cm)	2021CP	10.42	16.25	6.30–16.53	12.33 ± 1.62	0.20	0.58	13.16	0.60
	2021BJ	11.23	17.44	5.90–16.50	11.82 ± 1.60	–0.02	0.65	13.54	
	2021QS	11.52	16.43	11.66–16.86	11.57 ± 1.83	0.29	0.28	15.83	
	BLUE	11.22	16.81	2.35–10.97	12.02 ± 1.48	0.23	0.90	12.33	
AL-Top (cm)	2021CP	8.25	0	0–8.90	3.56 ± 2.95	0.21	–1.66	82.84	0.98
	2021BJ	8.76	0	0–9.09	3.49 ± 3.10	0.25	–1.71	88.97	
	2021QS	9.65	0	0.10–9.33	3.74 ± 2.98	0.26	–1.66	79.81	
	BLUE	8.84	0	0.09–8.87	3.57 ± 2.93	0.24	–1.72	82.21	
AL-Centre (cm)	2021CP	7.45	0	0–8.86	3.31 ± 3.33	0.24	–1.73	100.58	0.99
	2021BJ	7.56	0	0–9.59	3.27 ± 3.45	0.26	–1.75	105.42	
	2021QS	7.21	0	0–9.39	3.41 ± 3.37	0.26	–1.74	98.81	
	BLUE	7.48	0	0–9.18	3.30 ± 3.30	0.26	–1.75	100.04	
AL-Bottom (cm)	2021CP	5.87	0	0–6.85	2.17 ± 2.33	0.41	–1.47	107.04	0.97
	2021BJ	4.33	0	0–6.53	2.10 ± 2.33	0.40	–1.53	110.73	
	2021QS	4.34	0	0–7.76	2.58 ± 2.72	0.34	–1.64	105.56	
	BLUE	4.85	0	0–7.04	2.29 ± 2.43	0.35	–1.62	105.92	
SNS	2021CP	16	25	20.20–27.67	22.50 ± 1.84	–0.21	0.08	8.19	0.55
	2021BJ	17	24	15.80–27.40	23.54 ± 1.65	–0.01	–0.82	7.05	
	2021QS	17	26	15.30–27.40	22.01 ± 3.89	–0.40	0.09	17.69	
	BLUE	16.84	25	3.49–15.42	22.93 ± 1.48	–0.11	–0.41	6.46	
TGW (g)	2021CP	22.14	34.67	29.84–55.23	33.97 ± 6.03	0.08	0.80	17.76	0.77
	2021BJ	20.88	32.19	17.11–55.60	35.21 ± 5.55	0.01	1.20	15.77	
	2021QS	23.65	33.43	15.58–46.31	29.57 ± 5.93	0.41	0.16	20.05	
	BLUE	22.23	34.12	20.44–49.88	33.07 ± 4.31	0.19	0.73	13.02	
GL (mm)	2021CP	6.52	7.01	6.08–7.86	6.95 ± 0.36	0.05	–0.26	5.26	0.93
	2021BJ	6.33	7.28	6.37–8.06	7.15 ± 0.33	0.11	–0.31	4.67	
	2021QS	6.31	7.31	5.93–7.60	6.78 ± 0.34	0.01	–0.35	5.04	
	BLUE	6.38	7.24	6.26–7.80	6.96 ± 0.31	0.13	–0.19	4.4	
GW (mm)	2021CP	2.93	3.57	2.42–3.92	3.28 ± 0.21	–0.12	0.79	6.55	0.77
	2021BJ	2.25	3.61	2.68–3.81	3.35 ± 0.19	–0.51	1.33	5.67	
	2021QS	2.57	3.81	2.59–3.78	3.16 ± 0.21	0.12	0.21	6.88	
	BLUE	2.78	3.66	2.14–3.78	3.27 ± 0.16	–0.06	0.42	4.68	

Env: environment, BLUE: Best linear unbiased estimates, CV: coefcient of variation, *H*^*2*^: broad-sense heritability. XX: Xinxiang, ZX: Zhaoxian, CP: Changping, BJ: Beijing, QS: Qingshui

Variations were observed in traits of the Qingxinmai × 041133 RIL population investigated. The coefficients of variation (CV) of the tiller and spike traits (i.e., TN, PTN, SL, SNS, and AL) was greater than that of the grain traits (i.e., TGW, GL, and GW) (Table 1). A nearly normal frequency distribution of TN, PTN, SNS, SL, TGW, GL, and GW for the RILs was observed in each environment and the best linear unbiased estimate (BLUE) datasets with the absolute values of Skewness and Kurtosis coefficients approaching 0, except for TN and PTN obtained at 2020CP. (Table 1, Figure S1), suggesting the quantitative inheritance controlled by multiple loci. Awn lengths measured in the three positions on spikes showed a bimodal frequency distribution with the Kurtosis coefficients > 1, indicating the presence of major gene for these traits.

Correlation analysis demonstrated that the grain traits (TGW, GL, and GW) and the spike traits (SL and SNS) were positively correlated (*r* = 0.20–0.77, *P* < 0.01), but they were negatively correlated with TN and PTN (*r* = 0.14–0.55, *P* < 0.05) (Figure S2). TN and PTN were positively correlated with each other (*r* = 0.57, *P* < 0.01). Lengths of awn on the top, centre, and bottom of spikes were correlated (*P* < 0.01), and they were also correlated with GL, GW and SNS (*P* < 0.05). Except for TN, the other 7 traits were significantly correlated among different environments (*P* < 0.01) (Table S1). A significant interaction was observed between genotypes and environments for TN and PTN, while no such interaction was detected for the other traits (Table S2).

### BSE-Seq analysis

**Fig. 2:**
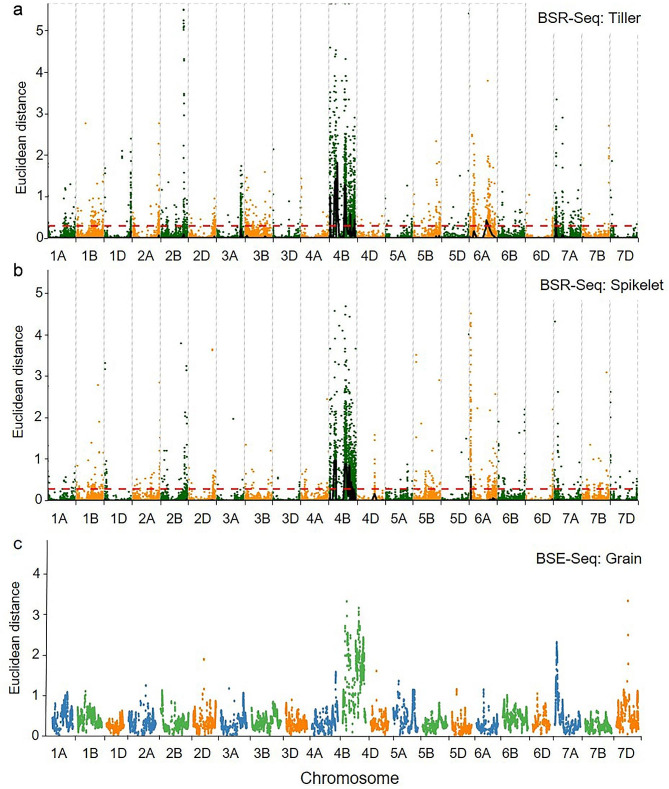
Distribution of single nucleotide polymorphisms (SNPs) between the phenotypically contrasting bulks of the Qingxinmai × 041133 RIL population. (**a**) BSR-Seq analysis of the high- and low-tiller number RNA pools (Bulk-HTN and Bulk-LTN); (**b**) BSR-Seq analysis of the long- and short-spike RNA pools (Bulk-LS and Bulk-SS); and (**c**) exon trapping analysis of the high- and low-thousand-grain weight pools (Bulk-HTGW and Bulk-LTGW)

The statistical parameters from the BSE-Seq analysis are summarized in Table S3. A total of 10,774 SNP variants were detected between the high- and low-TGW DNA pools (Bulk-HTGW and Bulk-LTGW). The most abundant enrichment of the TGW-associated SNPs and InDels was observed in the genomic intervals of 28.60–206.77 Mb and 342.32–621.14 Mb on chromosome 4B (8075) and 33.00–87.24 Mb on chromosome 7 A (2699) of Chinese Spring (CS) reference genome sequence RefSeq v1.0 (Fig. 2c, Table S3).

### BSR-Seq analysis of crowns and inflorescences

The statistical parameters of the RNA sequencing and comparisons between the RNA pools from contrasting tiller numbers (Bulk-HTN vs. Bulk-LTN) and between the RNA pools from contrasting spike lengths (Bulk-LS vs. Bulk-SS) are shown in Table S3. Raw reads of these RNA samples ranged from 135,474,852 to 156,857,352, and clean reads after trimming ranged from 129,072,674 to 149,787,196. The proportions of clean reads that were mapped to the CS reference genome RefSeq v1.0 were over 95%. The BSR-Seq analysis identified 1061 and 1508 SNP variants by comparing crown (Bulk-HTN vs. Bulk-LTN) and inflorescence (Bulk-LS vs. Bulk-SS) samples, respectively. Most SNP variants from tiller samples (Bulk-HTN vs. Bulk-LTN) were enriched on chromosome 4B (888), 6A (128), and 7A (45). SNPs from inflorescence samples (Bulk-LS vs. Bulk-SS) were mostly anchored on chromosomes 4B (1342), 4D (67), and 6A (99) (Fig. 2, Table S3). Most of the 464 common SNP variants detected in the tiller and inflorescence samples were located on chromosome 4B.

### Construction of the genetic linkage map

The 16 K GBTS SNP array applied for the RIL population of Qingxinmai × 041133 generated 37,699 original SNPs. After removing those with the coverage depth < 5×, 14,868 SNPs were retained. Polymorphic SNPs between parents Qingxinmai and line 041133 were 4,939 in 2,430 loci. A total of 2,398 loci with clear positions on the reference genome were used to construct a genetic linkage map (3113.1 cM). Thirty linkage groups were established. Most chromosomes had single linkage groups, except for two for chromosomes 1D, 3D, 5D, 6B, and 7D and three for chromosomes 6A and 6D. The marker density of this genetic linkage map was 3.27 cM per locus and 1.30 cM per marker. Subgenomes A, B, and D consisted of 344, 455, and 154 loci with the map distances of 1179.2, 1282.2, and 651.7 cM, respectively (Table S4). The genetic linkage maps constructed ranged from 50.9 cM (6D) to 197.3 cM (7A). Each chromosome contained 12 (4D) to 232 (3B) SNP markers. The average distance between adjacent SNPs and bin markers was in a range of 0.79 (3A) to 8.13 cM (3D) and 2.12 (2B) to 8.81 cM (3D). The marker orders on most chromosomes were generally consistent with those in the CS reference genome sequence RefSeq v1.0 (Figure S3).

The Chi-squared test revealed a genetic distortion in 377 polymorphic molecular markers (*P* < 0.05). Among them, 219 (58.09%) and 158 (41.91%) markers were biased towards line 041133 and Qingxinmai, respectively. Fifteen segregation distortion regions (SDR, ≥3SD loci) were detected in the RIL population. Eleven SDR originated from Qingxinmai and 4 from line 041133 (Table S5).

### QTL mapping

Sixty QTL for the 8 traits investigated were detected on 18 chromosomes, except for 5D, 6D, and 7B. Nine QTL were detected in multiple environments and the BLUE data sets, explaining phenotypic variations ranging from 3.43 to 65.34%. Fifteen QTL were detected in one or two environments and the BLUE data sets, explaining phenotypic variations ranging from 1.53 to 15.27%. The resting 36 QTL were observed in single environments, explaining 3.15–19.41% of the phenotypic variations (Table 2, Figure S4-S9).

**Table 2:** Quantitative trait loci (QTL) for total tiller number (TN), productive tiller number (PTN), spikelet number per spike (SNS), spike length (SL), awn length (AL), thousand-grain weight (TGW), grain length (GL), and grain width (GW) identified from different environments in the Qingxinmai × 041133 RIL population

QTLs	Env	Chr	Genomic interval (cM)	Flanking markers	LOD	PVE (%)	Add
*QTn.caas-1A*	2020ZX	1A	98.9–104.5	535,107,266–539,751,411	3.26	5.63	1.12
*QTn.caas-3B*	2020ZX	3B	24.7–28.3	40,776,427−44,857,581	3.03	5.46	–1.08
*QTn.caas-4B.1*	2020XX	4B	25.5–30.5	22,915,925−32,174,878	8.19	15.27	–1.35
	BLUE	4B	25.5–30.5	22,915,925−32,174,878	7.26	13.75	–0.91
*QTn.caas-4B.2*	2020ZX	4B	78.5–81.3	412,492,391−423,957,993	4.32	7.77	–1.31
*QTn.caas-5B.1*	2020CP	5B	73.7–76.8	541,684,640−545,791,979	4.71	9.12	–1.24
*QTn.caas-5B.2*	BLUE	5B	87.4–88.2	559,783,503–566,489,502	4.61	8.81	–0.65
*QTn.caas-7A*	BLUE	7A	190.9–194.6	724,015,398–728,083,088	4.05	7.68	–0.61
*QPtn.caas-2D*	2020XX	2D	126.7–127.7	608,762,484–612,343,111	3.10	4.11	0.86
	BLUE	2D	126.7–127.7	608,762,484–612,343,111	3.08	4.00	0.65
*QPtn.caas-4B*	2020XX	4B	25.5–30.5	22,915,925−32,174,878	18.07	28.97	–2.35
	2020ZX	4B	25.5–30.5	22,915,925−32,174,878	12.34	19.50	–2.32
	BLUE	4B	25.5–30.5	22,915,925−32,174,878	20.49	32.41	–1.92
*QPtn.caas-5A*	2020ZX	5A	95.4–96.2	504,711,920−523,864,171	2.81	3.98	–1.01
*QPtn.caas-5B*	2020BJ	5B	0–1.3	11,450,037−10,528,006	5.79	11.73	1.01
*QPtn.caas-7A*	2020ZX	7A	190.9–194.6	724,015,398–728,083,088	3.46	5.14	–1.15
*QSl.caas-1A*	2021QS	1A	57.9–58.2	396,862,371–431,983,495	2.88	4.01	–0.38
*QSl.caas-2A*	2021CP	2A	65.6–68.0	202,079,261–359,396,944	6.65	7.15	–0.48
	BLUE	2A	65.6–68.0	202,079,261–359,396,944	5.23	5.34	–0.36
*QSl.caas-2D*	2021CP	2D	99.8–104.7	540,218,193–577,340,696	3.31	3.43	0.33
	2021QS	2D	99.8–104.7	540,218,193–577,340,696	4.46	6.88	0.49
	BLUE	2D	99.8–104.7	540,218,193–577,340,696	4.05	4.08	0.31
*QSl.caas-4A*	2021BJ	4A	38.4–38.7	104,345,512–108,383,401	3.90	6.24	0.41
	2021CP	4A	38.4–38.7	104345512–108,383,401	4.60	4.89	0.39
	BLUE	4A	38.4–38.7	104345512–108,383,401	5.09	5.21	0.35
*QSl.caas-4B*	2021CP	4B	25.5–30.5	22,915,925−32,174,878	20.72	34.17	1.00
	2021BJ	4B	25.5–30.5	22,915,925−32,174,878	12.75	22.75	0.81
	2021QS	4B	25.5–30.5	22,915,925−32,174,878	13.93	25.51	0.97
	BLUE	4B	25.5–30.5	22,915,925−32,174,878	21.89	36.07	0.95
*QSl.caas-6A*	2021BJ	6A	12.6–20.0	605,342,228–616,822,113	3.04	5.01	–0.37
	BLUE	6A	12.6–20.0	605,342,228–616,822,113	3.17	3.26	–0.28
*QSns.caas-1B.1*	BLUE	1B	47.0–49.2	350,888,580−336,642,293	3.83	5.33	–0.31
*QSns.caas-1B.2*	2021QS	1B	179.2–184.8	670,413,689−677,093,559	5.20	9.17	0.68
*QSns.caas-1D*	2021QS	1D	15.3–18.0	18,152,685−20,050,138	2.81	4.62	0.48
*QSns.caas-2A*	2021CP	2A	65.6–68.0	202,079,261–359,396,944	3.96	5.26	–0.38
	BLUE	2A	65.6–68.0	202,079,261–359,396,944	3.13	4.46	–0.29
*QSns.caas-2D.1*	2021BJ	2D	53.7–68.0	76,747,629−175,764,113	3.05	7.29	0.44
	BLUE	2D	53.7–68.0	76,747,629−175,764,113	3.06	4.92	0.30
*QSns.caas-2D.2*	2021CP	2D	97.6–99.1	502,217,152–521,528,010	6.28	8.43	0.47
	BLUE	2D	97.6–99.1	502,217,152–521,528,010	4.41	6.19	0.34
*QSns.caas-3A.1*	2021CP	3A	98.2–100.0	670,666,314–676,809,677	3.78	5.04	–0.36
*QSns.caas-3A.2*	2021QS	3A	126.0–128.3	711,291,590–714,940,445	3.66	6.17	–0.56
*QSns.caas-4B*	2021CP	4B	25.5–30.5	22,915,925−32,174,878	3.47	6.70	0.47
	BLUE	4B	25.5–30.5	22,915,925−32,174,878	3.46	6.86	0.43
*QSns.caas-7D.1*	BLUE	7D	48.7–49.3	223,482,565−241,471,271	8.33	12.34	0.47
*QSns.caas-7D.2*	2021BJ	7D	64.8–67.6	410,525,843−427,004,562	3.61	8.64	0.47
	2021CP	7D	64.8–67.6	410,525,843−427,004,562	7.16	9.86	0.50
*QTgw.caas-2A.1*	2021CP	2A	25.5–34.3	35,633,343−42,499,829	2.68	4.22	0.04
*QTgw.caas-2A.2*	2021BJ	2A	50.8–52.1	58,328,036–59,582,849	4.84	8.79	0.06
*QTgw.caas-3D*	BLUE	3D	11.7–14.2	2,967,864–9,122,608	3.74	5.87	1.06
*QTgw.caas-4A.1*	2021CP	4A	38.4–38.7	104,345,512–108,383,401	2.68	4.18	–0.04
*QTgw.caas-4A.2*	2021BJ	4A	102.9–106.1	591,709,222–597,478,011	3.20	5.68	0.04
*QTgw.caas-4B*	2021CP	4B	25.5–30.5	22,915,925−32,174,878	8.13	13.94	0.09
	2021BJ	4B	25.5–30.5	22,915,925−32,174,878	3.62	6.47	0.06
	2021QS	4B	25.5–30.5	22,915,925−32,174,878	5.27	10.61	0.07
	BLUE	4B	25.5–30.5	22,915,925−32,174,878	14.77	25.30	2.32
*QTgw.caas-6B*	2021CP	6B	190.7–195.6	682,879,787−687,067,040	2.78	4.40	0.04
*QGl.caas-1A*	BLUE	1A	74.0–75.1	497,996,091–508,644,550	3.28	4.91	0.06
*QGl.caas-1B*	2021QS	1B	60.9–64.4	453,204,679−469,086,270	3.88	4.72	–0.08
	BLUE	1B	60.9–64.4	453,204,679−469,086,270	5.06	8.28	–0.08
*QGl.caas-2B*	BLUE	2B	124.5–125.6	742,472,704–747,180,146	3.92	5.88	–0.06
	2021CP	2B	124.5–125.6	742,472,704–747,180,146	3.81	5.86	–0.09
*QGl.caas-3A*	2021QS	3A	30.6–50.5	37,658,624−59,721,072	3.52	5.94	–0.09
*QGl.caas-3B*	2021BJ	3B	37.4–38.1	124,443,004–127,321,716	8.87	11.37	–0.11
	2021QS	3B	37.4–38.1	124,443,004–127,321,716	4.38	5.26	–0.08
	BLUE	3B	37.4–38.1	124,443,004–127,321,716	4.95	7.61	–0.07
*QGl.caas-4A*	2021BJ	4A	102.9–106.1	591,709,222–597,478,011	5.98	8.03	0.09
*QGl.caas-4B.1*	2021CP	4B	25.5–30.5	22,915,925−32,174,878	13.97	23.93	0.18
	2021BJ	4B	25.5–30.5	22,915,925−32,174,878	10.01	13.22	0.13
	2021QS	4B	25.5–30.5	22,915,925−32,174,878	10.69	14.12	0.14
	BLUE	4B	25.5–30.5	22,915,925−32,174,878	7.83	12.61	0.10
*QGl.caas-4B.2*	2021BJ	4B	78.5–81.3	412,492,391−423,957,993	10.35	19.41	0.15
*QGl.caas-4B.3*	2021CP	4B	91.3–93.4	473,334,694−485,824,533	7.12	14.64	0.12
	BLUE	4B	91.3–93.4	473,334,694−485,824,533	5.94	11.80	0.09
*QGl.caas-4D*	2021QS	4D	20.3–22.8	65,859,359–85,257,453	3.86	4.66	0.08
*QGl.caas-5A*	BLUE	5A	129.3–131.3	576,537,928−581,934,034	4.42	6.75	0.07
*QGl.caas-6A.1*	2021CP	6A	50.7–55.2	465,633,131–481,618,636	2.82	4.23	0.07
*QGl.caas-6A.2*	2021BJ	6A	0.3–9.1	598,763,730−607,425,711	2.66	3.28	0.06
*QGl.caas-6B*	2021BJ	6B	40.8–42.7	118,821,640−131,147,565	4.91	5.95	0.08
*QGl.caas-7A*	2021QS	7A	168.5–171.5	693,302,812−696,799,296	5.22	6.38	–0.09
*QGl.caas-7D*	2021BJ	7D	27.0–28.3	132,001,714−137,496,491	2.66	3.15	–0.06
*QGw.caas-2A*	2021CP	2A	50.8–52.1	58,328,036–59,582,849	2.76	4.84	1.29
	2021BJ	2A	50.8–52.1	58,328,036–59,582,849	3.80	5.88	1.40
*QGw.caas-3D*	2021QS	3D	11.7–14.2	2,967,864–9,122,608	2.56	5.23	1.35
	BLUE	3D	11.7–14.2	2,967,864–9,122,608	3.71	6.70	0.04
*QGw.caas-4A*	2021BJ	4A	102.9–106.1	591,709,222–597,478,011	5.72	8.86	1.71
*QGw.caas-4B*	2021CP	4B	25.5–30.5	22,915,925−32,174,878	9.41	16.30	2.72
	2021BJ	4B	25.5–30.5	22,915,925−32,174,878	6.43	11.55	2.14
	2021QS	4B	25.5–30.5	22,915,925−32,174,878	6.09	12.18	2.14
	BLUE	4B	25.5–30.5	22,915,925−32,174,878	9.17	16.35	0.07
*QGw.caas-6B*	2021CP	6B	190.7–195.6	682,879,787−687,067,040	2.73	4.88	1.29
*QGw.caas-7A*	2021QS	7A	54.8–64.2	80,137,719−94,209,681	4.17	9.06	1.78
*QAl.caas-4B*	2021CP	4B	25.5–30.5	22,915,925−32,174,878	3.99	2.74	0.60
	BLUE	4B	25.5–30.5	22,915,925−32,174,878	2.89	1.53	0.44
*QAl.caas-5A*	2021CP	5A	191.3–195.8	688,174,490–697,644,183	56.12	59.96	–2.75
	2021BJ	5A	191.3–195.8	688,174,490–697,644,183	62.37	62.20	–2.96
	2021QS	5A	191.3–195.8	688,174,490–697,644,183	57.00	61.15	–2.82
	BLUE	5A	191.3–195.8	688,174,490–697,644,183	71.09	65.34	–2.81

Note: PVE, phenotypic variation explained; LOD, logarithm of the odd; Add, additive effect. Positive and negative values indicate that alleles from line 041133 and Qingxinmai increase the trait values, respectively; and BLUE, best linear unbiased estimates. XX, Xinxiang; ZX, Zhaoxian; CP, Changping; BJ, Beijing; and QS, Qingshui

#### Tiller traits

Twelve QTL conferring TN and PTN were identified on chromosomes 1A, 2D, 3B, 4B, 5A, 5B, and 7A (Figure S4-S9). A major locus, *QTn.caas-4B.1/QPtn.caas-4B*, was localized in the same genetic interval on chromosome 4B. It explained 13.75–32.41% of the phenotypic variations for TN and PTN with logarithm of odds (LOD) values of 7.26–20.49. The positive alleles of this locus for more tillers were contributed by Qingxinmai. Another minor effective QTL, *QPtn.caas-2D* for PTN, contributed by line 041133, was observed in one environment (2020XX) and the BLUE datasets. It explained 4.00% and 4.11% of the phenotypic variations. The remaining 9 loci were detected in single environments and explained 3.98–11.73% of the phenotypic variations. Most of them were contributed by Qingxinmai, except for two loci (*QTn.caas-1A* and *QPtn.caas-5B*) by line 041133 (Table 2).

#### Spike and awn traits

Nineteen QTL for SL, SNS, and AL were identified on chromosomes 1A, 1B, 1D, 2A, 2D, 3A, 4A, 4B, 5A, 6A, and 7D (Figure S4-S9). A major locus, *QSl.caas-4B*/*QSns.caas-4B*, was detected on chromosome 4B in four environments and the BLUE datasets. This QTL was contributed by line 041133 and explained the phenotypic variations of SL and SNS by 6.7–36.07% (LOD = 3.46–21.89). *QSl.caas-2A*, *QSl.caas-2D*, *QSl.caas-4A*, *QSl.caas-6A* for SL, and *QSns.caas-2A*, *QSns.caas-2D.1*, and *QSns.caas-2D.2* for SNS were detected on different chromosomes in one or two environments and the BLUE datasets. They explained 3.26–8.43% of the phenotypic variations (LOD = 3.04 to 6.65). The remaining seven QTL for spike and awn traits were observed in single environments, accounting for 4.01–12.34% of the phenotypic variations (Table 2).

**Fig. 3:**
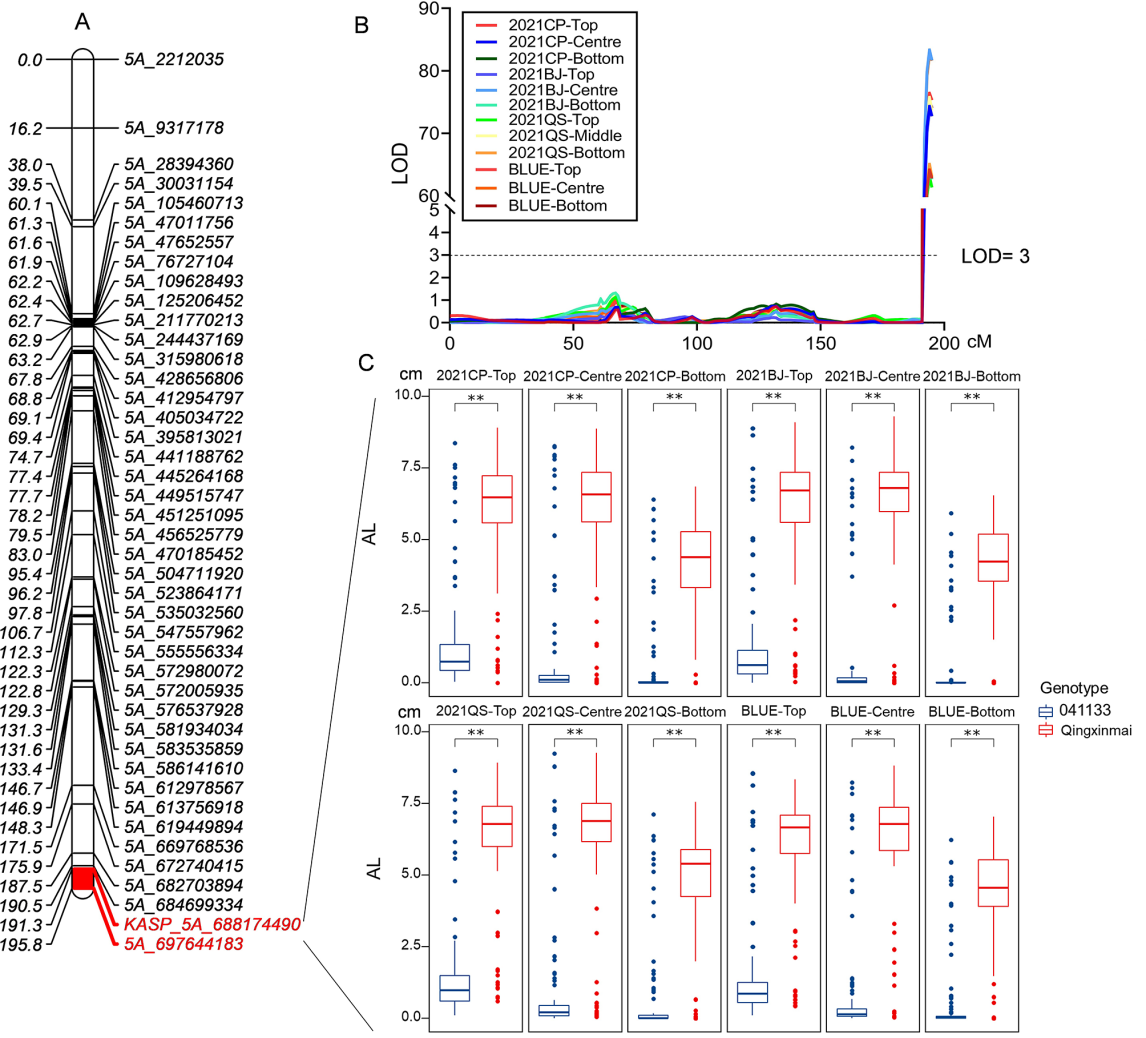
Genetic linkage map (**a**), QTL analysis method (**b**), and effects (**c**) of locus *QAL.caas-5A* on awn lengths. The BLUE values for awn lengths of the Qingxinmai × 041133 RILs were grouped based on the genotypes of the locus-specific KASP marker *KASP_5A_ 688174490*. **, *P* < 0.01

*QAl.caas-4B* for AL detected in 2021CP and the BLUE dataset was located in the same genetic interval as *QSl.caas-4B*/*QSns.caas-4B*. This QTL was contributed by 041133. But ALs on the top, central, and bottom of spikes appeared to be controlled by a major locus *QAl.caas-5A*. It was detected in all the three environments and the BLUE dataset, and explained the phenotypic variations of 59.96–65.34% (LOD = 56.12–71.09). The physical location of this locus was observed in a genomic interval of 688.17–697.64 Mb (Table 2). The additive effect of *QAl.caas-5A* was provided by Qingxinmai. The SNP sequence of the closest molecular marker *5A_688174490* obtained by the 16 K GBTS SNP array was converted to a Kompetitive allele-specific PCR (KASP) marker *KASP_ 5A_ 688174490* (Fig. 3, Table S6). This KASP marker proved to be tightly linked to *QAl.caas-5A* by genotyping the entire RIL population (Figure S10a).

#### Grain traits

Twenty-nine QTL for TGW, GW, and GL were detected on chromosomes 1A, 1B, 2A, 2B, 3A, 3B, 3D, 4A, 4B, 4D, 5A, 6A, 6B, 7A, and 7D (Figure S4-S9). Nine loci on chromosomes 1B, 2A, 2B, 3B, 3D, and 4B were identified in multiple environments. The major QTL, *QTgw/Gl/Gw.caas-4B* for the three grain traits, were detected in all environments and the BLUE datasets. This locus was contributed by line 041133 and explained 6.47-25.30% of the phenotypic variations (LOD = 3.62–14.77). Another five loci for grain weight, *QGl.caas-1B*, *QGl.caas-2B*, *QGl.caas-4B.3*, *QGw.caas-2A*, and *QGw.caas-3D*, were identified in two environments or the BLUE dataset, explaining 4.72–14.64% of the phenotypic variations. The favorable alleles of these QTL were also contributed by line 041133, except for *QGl.caas-1B* and *QGl.caas-2B* by Qingxinmai. *QGl.caas-3B* for GL on chromosome 3B were detected in two environments at sites 2021BJ and 2021QS and the BLUE datasets, explaining 5.26–11.37% of the phenotypic variations. The favorable allele of this locus was contributed by Qingxinmai. The remaining 20 minor QTL for the grain traits were detected in single environments, accounting for 3.15–19.41% of the phenotypic variations. Line 041133 contributed 16 positive alleles and Qingxinmai contributed the other 4 positive alleles (Table 2).

### Analysis of additive effects of the major QTL

**Fig. 4:**
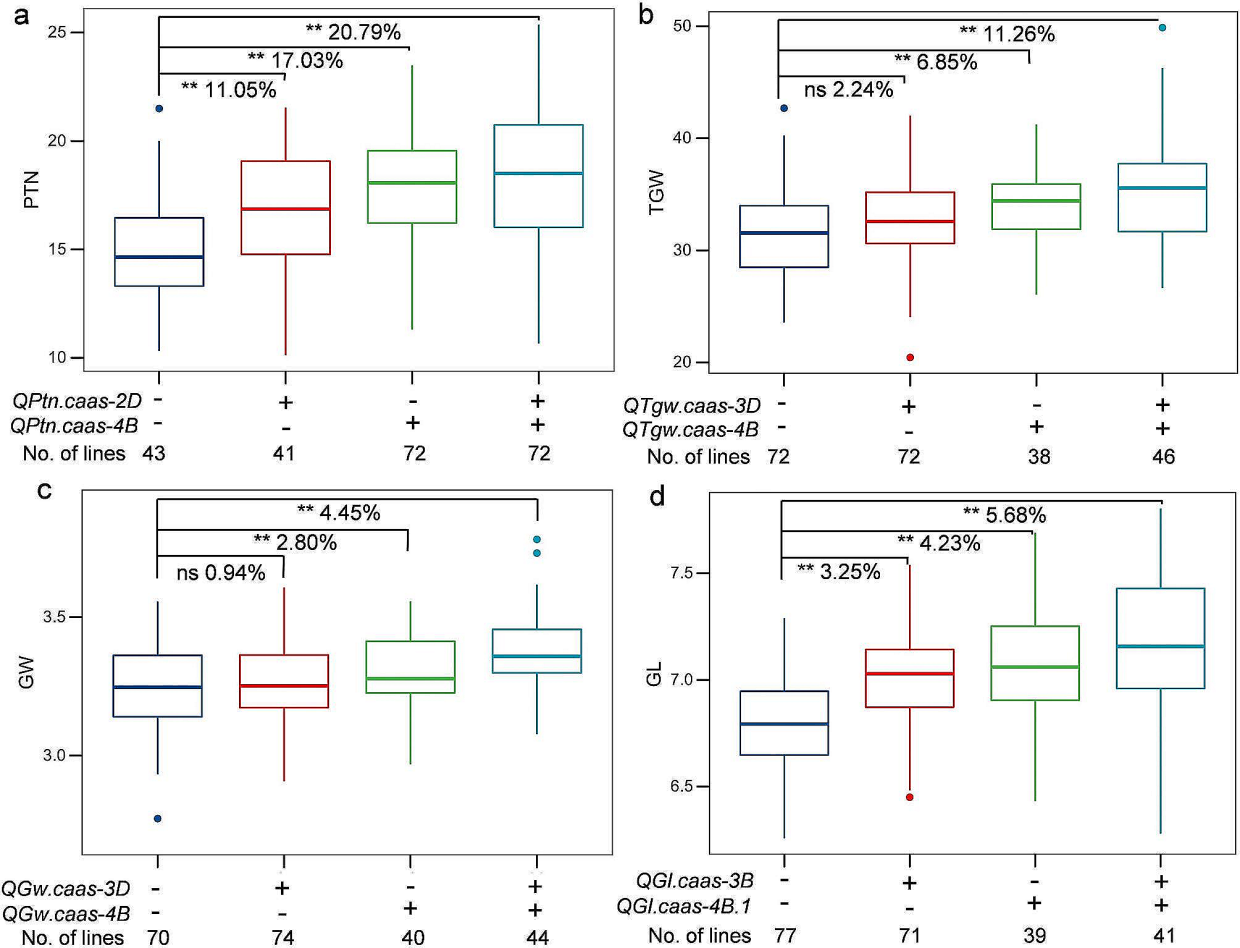
Additive effects of the QTL detected on productive tiller number (PTN) (**a**), thousand-grain weight (TGW) (**b**), grain width (GW) (**c**), and grain length (GL) (**d**) using the BLUE datasets of the Qingxinmai × 041133 RIL population. + and −: presence and absence of the favorable alleles of the target QTL based on genotypes of the flanking markers of the corresponding QTL. **, *P* < 0.01. ns: no significant difference

Since the pleiotropic QTL for PTN, SL, SNS, TGW, GW, and GL were detected on chromosome 4B, we analyzed the additive effects of this locus on the corresponding traits using the BLUE datasets of the mapping population. Lines with the favorable alleles of *QPtn/Sl/Sns/Tgw/Gl/Gw.caas-4B* only increased PTN, TGW, GL, and GW by 17.03%, 6.85%, 2.80%, and 4.23% over those without the alleles (Fig. 4). The addition of alleles at the minor loci *QPtn.caas-2D*, *QTgw.caas-3D*, *QGw.caas-3D*, and *QGl.caas-3B* further enhanced the performances of those traits by 20.79%, 11.26%, 4.45%, and 5.68%, respectively (Fig. 4). Lines with the favorable allele for SL at *QSl.caas-4B* only increased SL by 9.84% relative to lines without the favorable allele. The trait performances of SL and SNS appeared to be associated with the number of positive alleles. More favorable alleles increased the trait values by different magnitudes (Figure S11).

### Development of the KASP marker specific for *QTn/Ptn/Sl/Sns/Tgw/Gl/Gw.caas-4B*

**Fig. 5:**
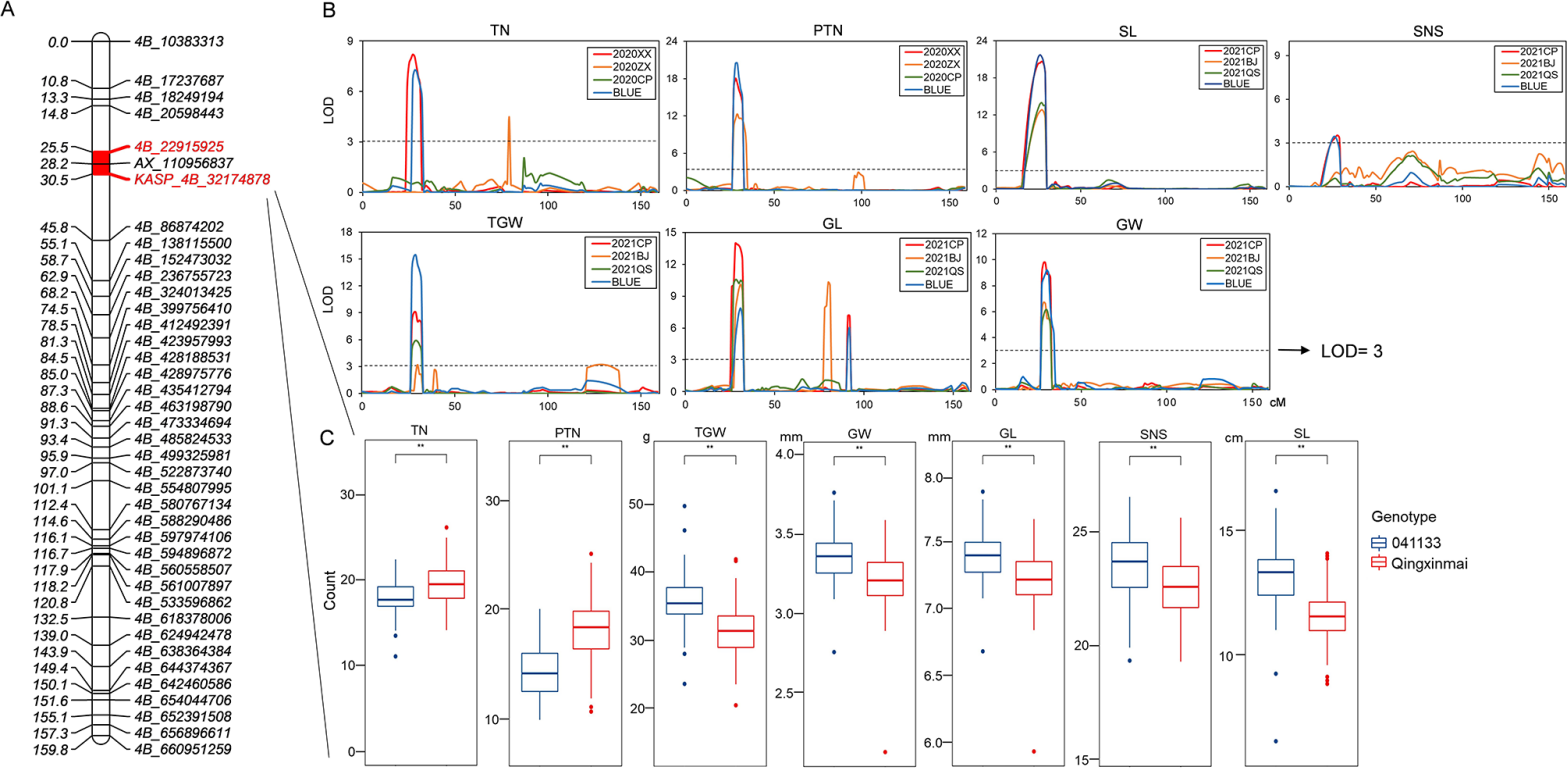
**a:** Genetic linkage map (**a**), QTL analysis method (**b**), and effects (**c**) of the pleiotropic locus *QTn/Ptn/Sl/Sns/Tgw/Gl/Gw.caas-4B* on total tiller number (TN), productive tiller number (PTN), spike length (SL), spikelet number per spike (SNS), thousand-grain weight (TGW), grain length (GL), and grain width (GW). The BLUE datasets of the Qingxinmai × 041133 RILs were grouped based on the genotypes of the locus-specific KASP marker *KASP_4B_32174878*. **, *P* < 0.01

Locus *QTn/Ptn/Sl/Sns/Tgw/Gl/Gw.caas-4B* for the tiller, spike, and grain traits was anchored in the physical interval of 22.92–32.17 Mb on chromosome 4B of the CS reference genome sequence RefSeq v1.0. A KASP marker, *KASP_4B_32174878*, was converted from the SNP locus *4B_32174878* (Table S6, Figure S10b). Two previously developed *QTkw.caas-4BS*-linked markers were also used to genotype Qingxinmai and line 041133. Marker *AX_89323611* exhibited monomorphism and the other marker *AX_110956837* was polymorphic. The two polymorphic markers *KASP_4B_32174878* and *AX_110956837* placed locus *QTn/Ptn*/*Sl*/*Sns*/*Tgw*/*Gl*/*Gw.caas-4B* in a physical interval of 3.23 Mb (28.95–32.17 Mb) in the CS reference genome RefSeq v1.0 (Fig. 5).

### Analysis of the annotated genes in the target genomic interval of *QTn*/*Ptn/Sl/Sns/Tgw/Gl/Gw.caas-4B*

We analyzed the genes in the genomic region where the QTL on chromosome 4B resides using the *in silico* expression method and the RNA-Seq data generated from the BSR-Seq analysis with crowns and inflorescences. Twenty-two high confidential genes were annotated in the genomic interval of the pleiotropic QTL on chromosome 4B of the Chinese Spring reference genome RefSeq v1.0 (Table S7). The *in silico* expression of these annotated genes was analyzed in the Hexaploid Wheat Expression Database (IWGSC Annotation v1.1) assembled in the Triticeae Multi-Omics Center (http://202.194.139.32/). Five genes *TraesCS4B01G042300* (pleckstrin homology domain), *TraesCS4B01G042900* (*ZnF*), *TraesCS4B01G043100* (*Rht-B1b*), *TraesCS4B01G043400* (phytanoyl-CoA dioxygenase), and *TraesCS4B01G044300* (microsomal glutathione S-transferase 3) were expressed in spikes and grains (Figure S12a). *TraesCS4B01G042900*, *TraesCS4B01G043100*, and *TraesCS4B01G043300* were also differentially expressed between Qingxinmai and line 041133 in the BSR-Seq analysis with the crown and inflorescence RNA samples (Figure S12b).

The expression of these genes in crowns and inflorescences of Qingxinmai and line 041133 were further determined by qPCR (Figure S13). Gene *TraesCS4B01G042900* was differentially expressed in crowns but not in inflorescences, *TraesCS4B01G043100* (*Rht-B1b*) in both tissues, and *TraesCS4B01G043300* in inflorescences but not in crowns. The expression of the three genes was higher in Qingxinmai than in line 041133, except *TraesCS4B01G043100* in crowns.

## Discussion

We detected a pleiotropic locus on chromosome 4B using a RIL population derived from a wheat landrace Qingxinmai and a breeding line 041133. We further determined that the alleles of this locus from the two parents confer different traits. The allele from Qingxinmai was responsible for increasing TN and PTN. The allele from line 041133 increased spike traits (SL and SNS) and grain traits (TGW, GL, and GW), and even had a minor effect on increasing AL. The effects of this locus on the traits investigated were enhanced by several minor effective QTL, such as *QPtn.caas-2D*, *QTgw.caas-3D*, *QGw.caas-3D*, and *QGl.caas-3B*.

Many QTL for various wheat plant growth and yield-related traits have been characterized with different mapping populations and various types of molecular markers [7, 32]. Wheat chromosome 4B was associated with tiller number [22, 33, 34], spike length and spikelet number [35–37], and grain weight in separate studies [8, 9, 36]. Yet some of those loci may not be localized in the same genomic regions of chromosome 4B. A QTL for tiller number was localized at 482.82 Mb [33]. Four loci for PTN were located on the genomic regions of 75.74-640.97 Mb [34, 38]. Liu et al. [38] and Deng et al. [39] identified a major QTL for PTN at 256.31 Mb. The allele of *QTn*/*Ptn.caas-4B* from Qingxinmai was anchored in the 28.95–32.17 Mb genomic region, which appears to be different from the above loci for tiller number. But the genomic location of locus *QTn*/*Ptn.caas-4B* overlaps with *QPtn.sau-4B* (28,941,377 and 32,167,076 bp) on chromosome 4B [22]. *QPtn.sau-4B* was detected in Chuannong 16, a spring wheat cultivar developed in Sichuan province, which is unrelated to Qingxinmai.

Wheat spike traits are also associated with locus on chromosome 4BS. *QSL.caas-4BS* was mapped to a physical position of 25.80–46.60 Mb in the Linmai 2 × Zhong 892 RIL population [36]. The association of chromosome 4B and grain weight was reported in a RIL population of Doumai/Shi 4185 [36]. In that study, one of the 11 QTL for grain weight *QTkw.caas-4BS* explained a high range of phenotypic variation (12.1–45.6%). That locus was located in a genomic region of 25.80–46.60 Mb. A 483-kb deletion in this region in Doumai contains genes *ZnF*, *EamA*, and *Rht-B1* [8]. In a most recent study, Song et al. [9] reported that wheat cultivar Heng 597 possessed a locus *QTgw.cau-4B* for grain weight. The deletion of approximately 500 kb fragment, also carrying these three genes, increased grain weight. The knockdown of *Rht-B1b* in Fielder increases plant height, spike length, and grain weight. But deletion of *ZnF-B* led to a slight reduction in grain size and plant height with no change in spike length compared to the wild-type Fielder. There is no large fragment deletion in the same genomic interval in Qingxinmai and line 041133 as in Doumai and Heng 597. Further study is needed to characterize the genes associated with the pleiotropic locus on chromosome 4B in the current study.

Selection for awns with minimal extension, colloquially referred to as ‘awnletted’, has been dominated by genes *Tipped1* (*B1*), *Tipped2* (*B2*), and *Hooded* (*Hd*). These loci inhibit awn development of wheat [40, 41]. We detected a major effective locus *QAl.caas-5A* for awn inhibition in the tip-awned line 041133. The target interval of this locus was located on chromosome 5A (688.17–697.64 Mb), which overlaps gene *B1* [42, 43]. *B1* inhibits awn elongation by impeding cytokinin- and auxin-promoted cell division and directly repressing the expression of *TaRAE2* and *TaLks2* [44]. Based on the genomic locations, *QAl.caas-5A* is most likely identical to gene *B1*.

Segregation distortion of molecular markers is present in different genetic populations. Paillard et al. [45] detected 17% segregation distortion of RFLP and SSR markers in the RILs from cross between Arina and Forno wheats. We detected two large SDRs, one on chromosome 2B from Qingxinmai, and the other on chromosome 6B from line 041133. Two SDRs, SDR-4B.2 (26.9–30.8 cM) and SDR-4B.3 (30.8–34.4 cM) from Qingxinmai, were associated with the pleiotropic locus *QPtn.caas-4B* for PTN. The SDRs may arise from chromosome recombination, gametophyte lethal genes, and segregation distortion factors [46, 47].

## Materials and methods

### Plant materials

Qingxinmai, a wheat landrace from Xinjiang, China, is characterized as long awn, slender spike, grain, and culm, and plenty of tillers (Fig. 1a). Line 041133 (pedigree: Jining 13/Tongmai 2) was developed in Qinghai province, with characteristics of tip-awns, thick spike, strong culm, fewer tillers, and larger grains (Fig. 1b). A RIL population consisting of 228 F_2:9_ lines was developed by a single seed decent method from cross Qingxinmai × 041133 to be used as the mapping population.

### Phenotype assessments

During the 2019–2020 and 2020–2021 wheat growing seasons, field plots were set for assessing traits of the mapping population and the parents in the experimental farms of Institute of Crop Sciences, Chinese Academy of Agricultural Sciences in Beijing (2021BJ, 116.33°E, 39.96°N) and Changping, Beijing (2020CP, 2021CP, 116.26°E, 40.17°N), Zhaoxian, Hebei province (2020ZX, 114.78°E, 37.75°N), and Xinxiang, Henan province (2020XX, 113.98°E, 35.32°N), as well as a farm of Gansu Academy of Agricultural Sciences in Qingshui, Gansu province (2021QS, 105.80°E, 34.60°N). About 40 seeds of each line were planted in a one-row plot 2.0 m in length and a row spacing of 30 cm. A randomized complete block design with two replicates was used to arrange the RILs and the parents in each site. Field managements were performed according to the local practices for wheat production. Tiller traits, including TN at the tillering stage [Zadocks growth stage (GS) 31] [48] and PTN at the late milk stage (GS 77) were enumerated in 10 plants from each plot. At maturity, ten plants were randomly harvested from each plot to measure SL from the base of the rachis to the tip of terminal spikelet excluding awns and enumerate SNS. Length of awns at the top, central, and bottom of five spikes were measured. The phenotypic data of TGW, GL, and GW were measured by the Wanshen SC-G Automatic Seed Testing Analysis and Thousand Grain Weight Software (WSeen Inc., Hangzhou, China).

### BSE-Seq analysis

Genomic DNA was isolated from grains with a cetyltrimethylammonium bromide method [49]. Bulked DNA pools (Bulk-HTGW and Bulk-LTGW) were constructed by separately mixing equal amounts of DNA samples from 40 high-TGW (36.5–49.9 g) and 40 low-TGW (20.4–29.0 g) RILs. These DNA bulks, together with the parents, were subjected to exome capture sequencing on the WheatPanExomeV2 platform at Chengdu Teuni Technology (Chengdu, China). Uncaptured DNA fragments were removed, and the enriched exons were amplified by PCR. High-throughput DNA sequencing was performed on the Illumina platform (Illumina Inc., San Diego, CA, USA). SNPs obtained by mutation detection were filtered with the criteria of allele frequency < 0.3 or > 0.7 using the SNP-index algorithm to determine the genotype frequency of the extreme bulks [50]. Significantly different SNP sites between the contrasting DNA bulks were statistically screened. Euclidean distance (ED) values of SNPs between the two DNA bulks were calculated using each allele depth with the quantile method. The ED values of SNP exceeding 99% was selected as the filtering threshold [51].

### BSR-Seq analysis

Crown and inflorescence were sampled at GS 24 and GS 31 for RNA sequencing. Thirty phenotypically contrasting RILs each were chosen based on phenotypes of tiller and spike traits to construct high- and low-tiller number (Bulk-HTN and Bulk-LTN) and long- and short-spike (Bulk-LS and Bulk-SS) pools. A BSR-Seq analysis was performed following a previously described pipeline [52]. In brief, RNA purified from the bulked samples with an Illumina TruSeq RNA sample preparation kit was sequenced on an Illumina Hiseq 4000 platform (Illumina Inc., San Diego, CA, USA). Adapter and low-quality sequences were truncated using Trimmomatic v0.36 [53]. High-quality reads were aligned against the Chinese Spring (CS) reference genome sequence RefSeq v1.0 (http://wheat-urgi.versailles.infa.fr) with the aid of STARv2.5.1b software [54]. Single nucleotide polymorphism (SNP) variants [*P* < 1e-8 for the Fisher’s Exact Test (FET) and the allele frequency difference (AFD) > 0.6] were identified from confident alignments using the “Haplotype Caller” module assembled in software GATK v3.6 [55].

### QTL mapping

The RILs and their parents were genotyped with genomic DNA samples by the wheat 16 K GBTS SNP array (MolBreeding Biotechnology Co. Ltd., Shijiazhuang, China) (http://www.molbreeding.com). Raw reads generated were processed with fastp v0.20.0 [56] and then aligned to the CS reference genome RefSeq v1.0 with Burrows-Wheeler aligner [57]. High-quality SNPs were obtained and filtered by GATK v3.5 [58], and SNP variants with the read depth < 5 were excluded from further analysis. SNP loci were classified as heterozygous genotypes when the SNP variation frequency ranged from 0.2 to 0.8, and the remaining genotypes were homozygous. Polymorphic SNPs between Qingxinmai and line 041133 were extracted for further analysis. A genetic linkage map was constructed for QTL calling using IciMapping 4.2 [59]. Only one marker was selected as a delegate from each bin to construct the linkage map. QTL for the traits were detected by IciMapping 4.2 with the inclusive composite interval mapping (ICIM) method. A test of 1,000 permutations was used to identify the logarithm of odds (LOD) threshold (> 3.0) that corresponded to a genome-wide false discovery rate of 5% (*P* < 0.05).

### KASP marker development and QTL validation

DNA sequences of selected SNPs were used to develop KASP markers with the help of the Triticeae Multi-omics Center (http://202.194.139.32). Primers were designed using PolyMarker (http://polymarker.tgac.ac.uk/). The probe sequences for the FAM and the HEX signals were separately added to the primers specific for the two parental genotypes. A thermal Cycler (C1000 Touch™, Bio-Rad, Foster City, CA, USA) was used to perform KASP assays. The reaction mixture (10 µl) was prepared by mixing 5 µl of 2× master mix (Wuhan Gentides Biotech Co., Ltd., Wuhan, China), 0.2 µl of primer mix, 3 µl of ddH_2_O, and 2 µl of DNA template (50–150 ng/µl). The thermal cycling profile included 94 °C for 15 min hot-start activation, a touchdown phase of 10 cycles (94 °C for 20 s, touchdown at 61 °C initially and then decreased by 0.6 °C per cycle for 60 s), and 26 cycles of regular PCR (94 °C for 20 s, 55 °C for 1 min). The following cycling and resting steps set at 94 °C for 20 s and 57 °C for 60 s (3–10 cycles per step) were performed if signals were poorly clustered. End-point fluorescence data were screened using the microplate reader FLUOstar Omega SNP (BMG Labtech, Durham, NC, USA) and analyzed by the Klustering Caller Software (http://www.lgcgroup.com/). The KASP markers polymorphic between the two parents were used to genotype the entire RIL population.

### Statistical analysis

The BLUE value was calculated with the Aov [analysis of variance (ANOVA) of multi-environmental trials] function in the QTL IciMapping 4.2 [59] to be used for combined QTL detection, correlation, and normal distribution analyses. The *H*^*2*^ for each trait were was also analyzed through QTL IciMapping 4.2. Phenotypic correlation was computed from the BLUE value of each line in SPSS v. 20.0 for Windows (IBM SPSS, Armonk, NY, USA). The Student’s *t*-test was used to evaluate the significance of differences in SPSS at *P* < 0.05 or *P* < 0.01.

### Expression of candidate genes in the target genomic region

Information on gene annotation in target genomic intervals was obtained with JBrowse in the Triticeae Multi-Omics Center (http://202.194.139.32). Gene codes annotated were used to predict the gene expression levels in the GeneExpression toolbar in the Triticeae Multi-Omics Center (http://202.194.139.32). Significantly differential expression was defined as an absolute log_2_ value (fold change) > 1 at *P* < 0.01.

Total RNA was isolated from crowns (sampled at GS 31) and inflorescences (sampled at GS 41) of Qingxinmai and line 041133 by a FastPure Universal Plant Total RNA Isolation Kit (Vazyme Biotech Co., Ltd., Nanjing, China). The first-strand cDNA was synthesized using a PrimeScript RT Reagent Kit with gDNA Eraser (https://www.takarabiomed.com.cn/). Gene-specific quantitative real-time PCR (qPCR) primer pairs for candidate genes were designed according to the gene annotations from the CS reference genome RefSeq v1.0 [60]. qPCR assays were performed on a BioRad CFX system with the Taq Pro Universal SYBR qPCR Master Mix (Vazyme Biotech Co., Ltd., Nanjing, China). Wheat *Actin* gene was amplified as the reference gene. Relative expression was determined using the 2^−ΔΔCT^ method [61]. Three biological replicates were taken from crowns and inflorescences. Three replicates for each RNA sample were run as technical replicates.

### Electronic supplementary material

Below is the link to the electronic supplementary material.

Supplementary Material 1

Supplementary Material 2

Figure S1

Figure S2

Figure S3

Figure S4

Figure S5

Figure S6

Figure S7

Figure S8

Figure S9

Figure S10

Figure S11

Figure S12

Figure S13

## Data Availability

The BSR-Seq and BSE-Seq data presented in this study are deposited in the National Genomics Data Center repository (https://ngdc.cncb.ac.cn/gsa/browse/CRA013836 and https://bigd.big.ac.cn/gsa/browse/CRA013931), accession number: CRA013836 and CRA013931.

## Abbreviations

AFD: The allele frequency difference
AL: Awn length
Aov: Analysis of variance (ANOVA) of multi-environmental trials
*B1*: *Tipped1*
*B2*: *Tipped2*
BJ: Beijing
BLUE: The best linear unbiased estimate
BSE-Seq: Bulked segregant exome capture sequencing
BSR-Seq: Bulked segregant RNA sequencing
Bulk-HTGW: the high-TGW DNA pool
Bulk-HTN: The high tiller numbers RNA pool
Bulk-LS: The long-SL RNA pool
Bulk-LTGW: The low-TGW DNA pool
Bulk-LTN: The low tiller numbers RNA pool
Bulk-SS: The short-SL RNA pool
CP: Changping
CS: The Chinese Spring
CTAB: Cetyltrimethylammonium bromide
CV: The coefficients of variation
ED: Euclidean Distance
FET: The Fisher’s Exact Test
GBTS: Genotyping by target sequencing
GL: Grain length
GNS: Grain numbers per spike
GW: Grain width
*H*^*2*^: The broad-sense heritability
*Hd*: *Hooded*
ICIM: The inclusive composite interval mapping
KASP: Kompetitive allele-specific PCR
LOD: The logarithm of odds
PTN: Productive tiller number
qPCR: Quantitative real-time PCR
QS: Qingshui
QTL: Quantitative trait loci
RIL: Recombinant inbred line, SDR: The segregation distortion regions
SL: Spike length
SN: Spikelet number
SNP: Single nucleotide polymorphism
SNS: Spikelet number per spike
TGW: Thousand-grain weight
TN: Tiller number
XX: Xinxiang
ZX: Zhaoxian
