# Supplementary material for: Alleles on locus chromosome 4B from different parents confer tiller number and the yield-associated traits in wheat
Source: BMC Plant Biol. 2024 May 24;24:454. doi: 10.1186/s12870-024-05079-4 (PMC11127307; doi:10.1186/s12870-024-05079-4)
Supplement: Supplementary file 2 — Supplementary Material 2 [file 12870_2024_5079_MOESM2_ESM.docx]

**Supplementary Information**

**Figure S1.** Distributions of tiller number (TN), productive tiller number (PTN), spikelet number per spike (SNS), spike length (SL), awn length (AL), thousand-grain weight (TGW), grain length (GL), and grain width (GW) for the RIL population of Qingxinmai × 041133. TN and PTN in the 2019-2020 wheat growing seasons; SNS, SL, AL, TGW, GL and GW in the 2020-2021 wheat growing seasons. XX, Xinxiang; ZX, Zhaoxian; CP, Changping; BJ, Beijing; and QS, Qingshui. ns, no significant difference; *, *P* < 0.05, and **, *P* < 0.01.

**Figure S2.** Distributions and correlation coefficients of tiller number (TN), productive tiller number (PTN), spikelet number per spike (SNS), spike length (SL), awn length (AL), thousand-grain weight (TGW), grain length (GL), and grain width (GW) for the RIL population of Qingxinmai × 041133 RILs based on the BLUE datasets. *, *P* < 0.05, and **, *P* < 0.01.

**Figure S3.** Loci distribution of the 16 K GBTS SNP array. The size of 500 kb window, and the CS v1.0 reference genome.

**Figure S4.** Distribution of the detected QTL for tiller number (TN), productive tiller number (PTN), spikelet number per spike (SNS), spike length (SL), awn length (AL), thousand-grain weight (TGW), grain length (GL), and grain width (GW) of the Qingxinmai × 041133 RILs on the 1A (**a**), 1B (**b**), and 1D (**c**) linkage groups. Supported intervals for QTL are indicated by vertical bars. Different colors of bars represent different QTL.

**Figure S5.** Distribution of the detected QTL for tiller number (TN), productive tiller number (PTN), spikelet number per spike (SNS), spike length (SL), awn length (AL), thousand-grain weight (TGW), grain length (GL), and grain width (GW) of the Qingxinmai × 041133 RILs on the 2A (**a**), 2B (**b**), and 2D (**c**) linkage groups. Supported intervals for QTL are indicated by vertical bars. Different colors of bars represent different QTL.

**Figure S6.** Distribution of the detected QTL for tiller number (TN), productive tiller number (PTN), spikelet number per spike (SNS), spike length (SL), awn length (AL), thousand-grain weight (TGW), grain length (GL), and grain width (GW) of the Qingxinmai × 041133 RILs on the 3A (**a**), 3B (**b**), and 3D (**c**) linkage groups. Supported intervals for QTL are indicated by vertical bars. Different colors of bars represent different QTL.

**Figure S7.** Distribution of the detected QTL for tiller number (TN), productive tiller number (PTN), spikelet number per spike (SNS), spike length (SL), awn length (AL), thousand-grain weight (TGW), grain length (GL), and grain width (GW) of the Qingxinmai × 041133 RILs on the 4A (**a**), 4B (**b**), and 4D (**c**) linkage groups. Supported intervals for QTL are indicated by vertical bars. Different colors of bars represent different QTL.

**Figure S8.** Distribution of the detected QTL for tiller number (TN), productive tiller number (PTN), spikelet number per spike (SNS), spike length (SL), awn length (AL), thousand-grain weight (TGW), grain length (GL), and grain width (GW) of the Qingxinmai × 041133 RILs on the 5A (**a**), 5B (**b**), and 6A (**c, d**) linkage groups. Supported intervals for QTL are indicated by vertical bars. Different colors of bars represent different QTL.

**Figure S9.** Distribution of the detected QTL for tiller number (TN), productive tiller number (PTN), spikelet number per spike (SNS), spike length (SL), awn length (AL), thousand-grain weight (TGW), grain length (GL), and grain width (GW) of the Qingxinmai × 041133 RILs on the 6B (**a**), 7A (**b**), and 7D (**c**) linkage groups. Supported intervals for QTL are indicated by vertical bars. Different colors of bars represent different QTL.

**Figure S10.** KASP assays of markers *KASP_5A_ 688174490* specific for the locus *QAL.caas-5A* conferring awn lengths (**a**) and *KASP_4B_32174878* specific for the pleiotropic locus *QTn/Ptn*/*Sl*/*Sns*/*Tgw*/*Gl*/*Gw.caas-4B* (**b**).

**Figure S11.** Additive effects of three spike length (SL)-related QTL (**a**) and six spikelet number per spike (SNS)-related QTL (**b**) on SL and SNS based on the BLUE datasets. + and −: presence or absence of the favorable alleles of the target loci as detected by the flanking markers of the corresponding QTL, respectively. ns: no significant difference; *: *P* < 0.05; **: *P* < 0.01.

**Figure S12.** The *in silico* expression of 22 annotated genes of the pleotropic locus *QTn/Ptn*/*Sl*/*Sns*/*Tgw*/*Gl*/*Gw.caas-4B* in the target interval as revealed in the Triticeae Multi-Omics Center (http://202.194.139.32/) (a) and the BSR-Seq analysis using the crown and inflorescence samples (b).

**Figure S13.** qPCR analysis of the candidate genes *TraesCS4B01G042900*, *TraesCS4B01G043100*, and *TraesCS4B01G043300* on the crowns and inflorescences of Qingxinmai and line 041133. Bars indicate standard variation (n = 6). ns: no significant difference, *: *P* < 0.05, **: *P* < 0.01.

**Table S1** Correlation analysis between different trait environments. ns: no significant difference; *: *P* < 0.05; and **: *P* < 0.01.

**Table S2** Analysis of variance of traits (TN, PTN, SL, SNS, TGW, GL, GW, AL) for 228 RILs derived from the 041133×Qingxinmai cross. ns: no significant difference; *: *P* < 0.05; and **: *P* < 0.01.

**Table S3** Statistical Table of BSR and BSE Sequencing Results.

**Table S4** Distribution of markers and marker density across chromosomes in the common wheat map developed in Qingxinmai×041133 RILs population.

**Table S5** Distribution of segregation distortion regions in molecular linkage map.

**Table S6** Newly designed KASP markers specific for *QTn/Ptn/Sl/Sns/Tgw/Gl/Gw.caas-4B* and *QAl.caas-5A* and qPCR primers.

**Table S7** Gene annotations within *QTn/Ptn/Sl/Sns/Tgw/Gl/Gw.caas-4B* the genomic interval in the Chinese Spring reference genome RefSeq v1.0.
