# Supplementary figures and images for: Alleles on locus chromosome 4B from different parents confer tiller number and the yield-associated traits in wheat
Source: BMC Plant Biol. 2024 May 24;24:454. doi: 10.1186/s12870-024-05079-4 (PMC11127307; doi:10.1186/s12870-024-05079-4)

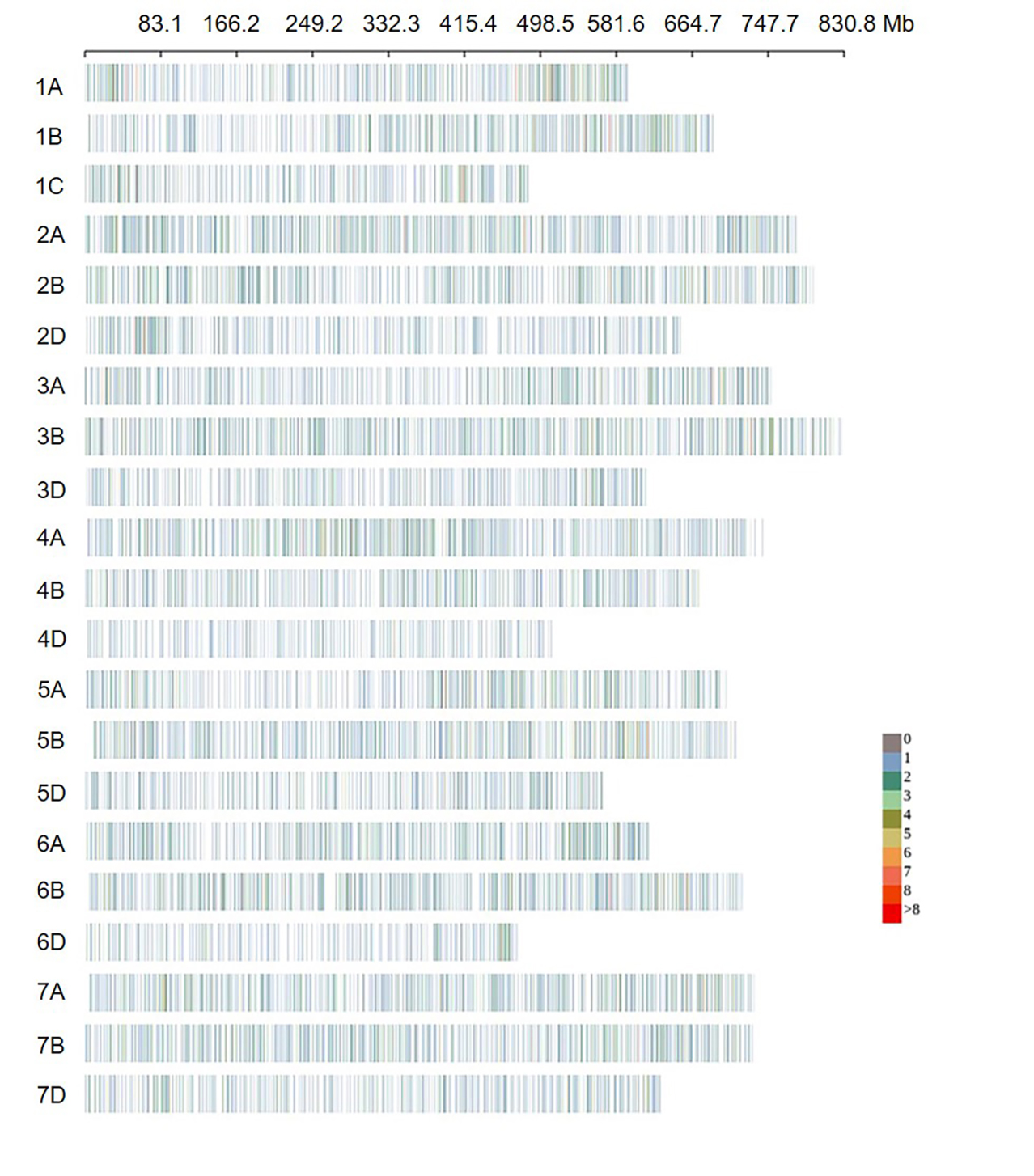

Supplement: Supplementary file 5 — Figure S3 [file 12870_2024_5079_MOESM5_ESM.jpg]

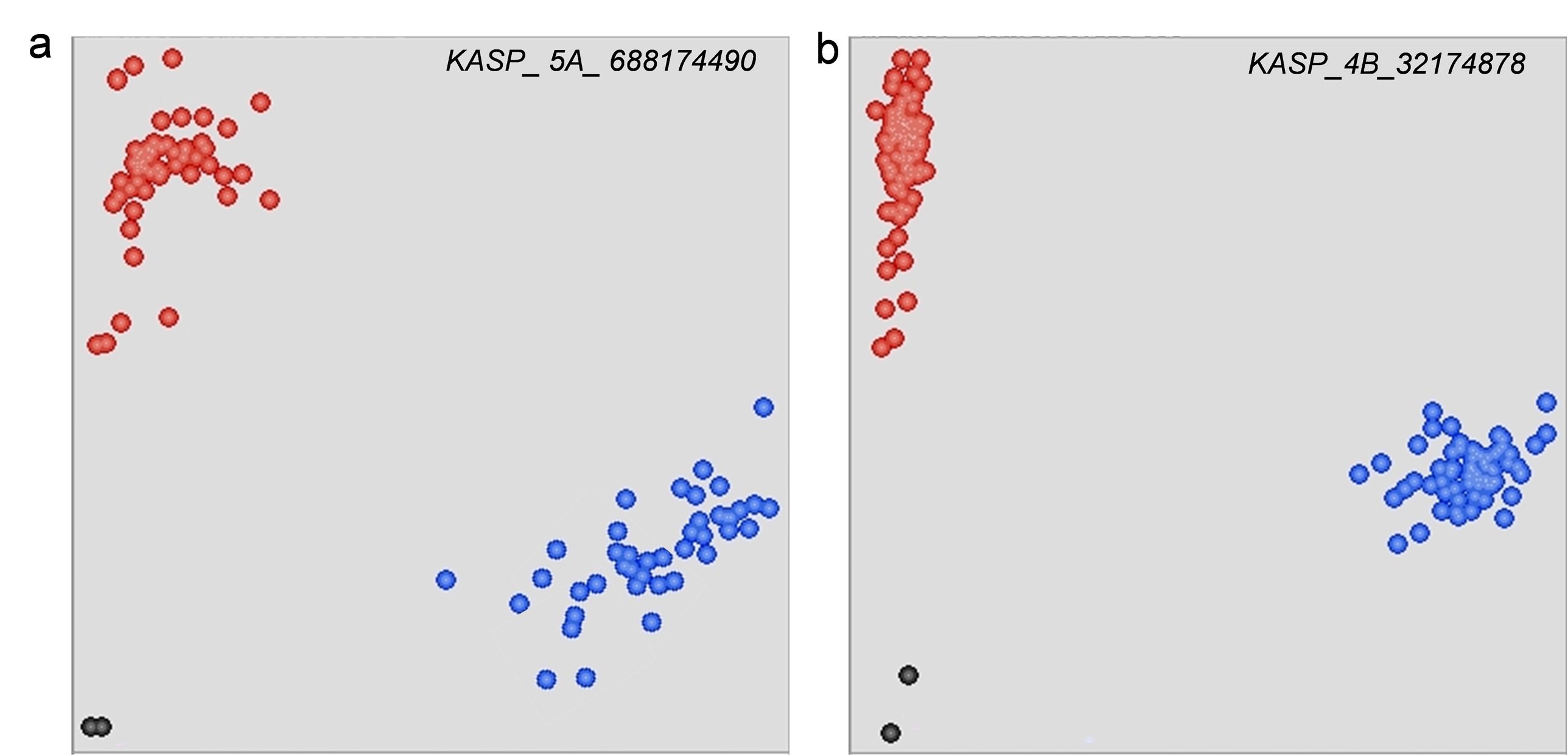

Supplement: Supplementary file 12 — Figure S10 [file 12870_2024_5079_MOESM12_ESM.jpg]

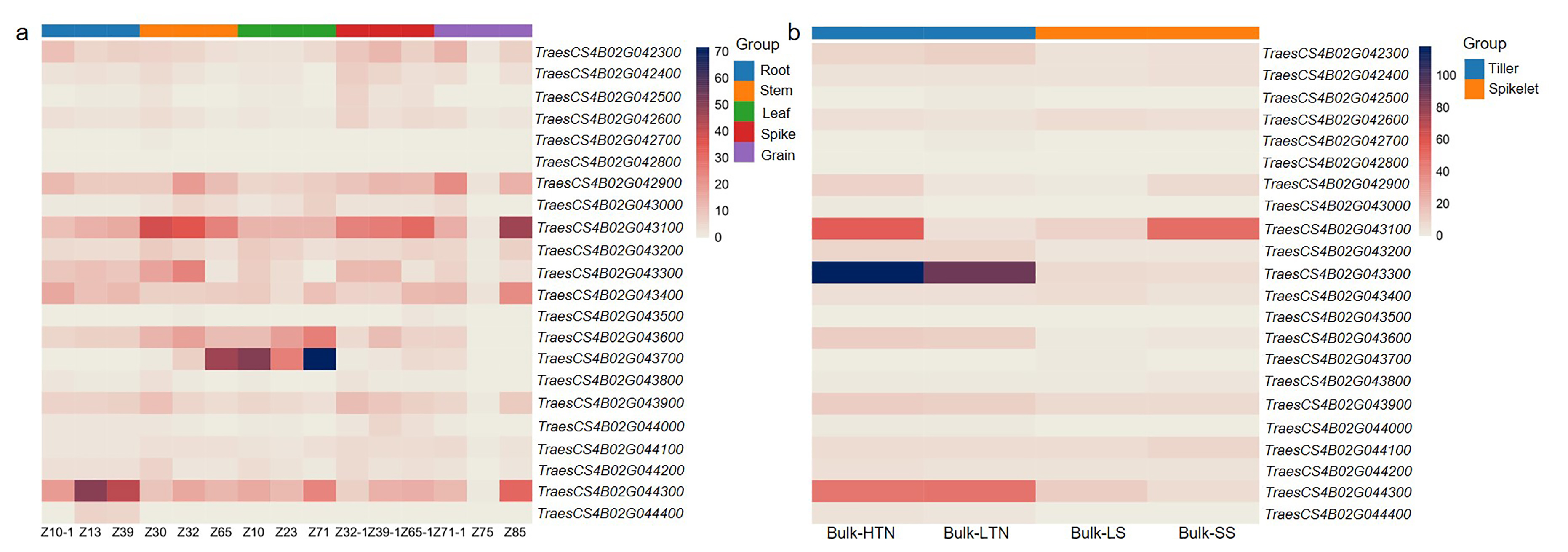

Supplement: Supplementary file 14 — Figure S12 [file 12870_2024_5079_MOESM14_ESM.jpg]

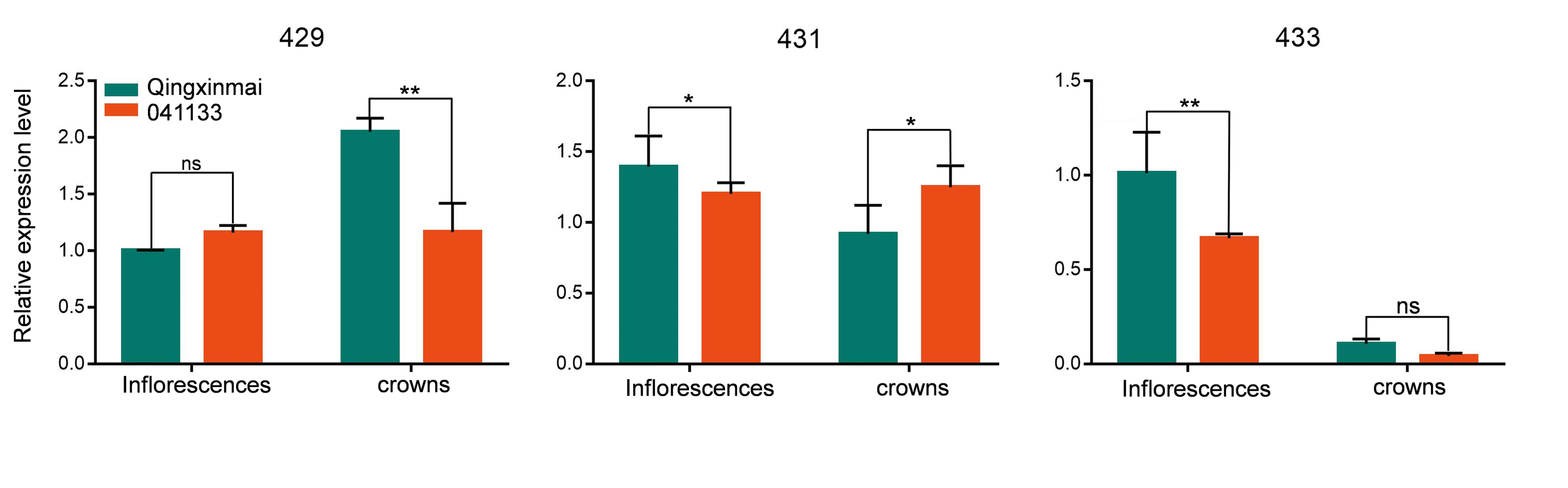

Supplement: Supplementary file 15 — Figure S13 [file 12870_2024_5079_MOESM15_ESM.jpg]
